# Characterization of Colombian Clay and Its Potential Use as Adsorbent

**DOI:** 10.1155/2018/5969178

**Published:** 2018-10-24

**Authors:** Iván Fernando Macías-Quiroga, Gloria Inés Giraldo-Gómez, Nancy Rocío Sanabria-González

**Affiliations:** ^1^Departamento de Ingeniería Química, Facultad de Ingeniería y Arquitectura, Universidad Nacional de Colombia Sede Manizales, Campus La Nubia, Km 7 Vía al Aeropuerto, AA 127, Manizales, Colombia; ^2^Departamento de Física y Química, Facultad de Ciencias Exactas y Naturales, Universidad Nacional de Colombia Sede Manizales, Campus La Nubia, Km 7 Vía al Aeropuerto, AA 127, Manizales, Colombia

## Abstract

This paper presents a mineralogical and physicochemical characterization of a Colombian clay found in an area with the greatest exploitation potential of smectites and possible use as an adsorbent for the removal of chromium. The clay was characterized by using X-ray diffraction (XRD), cation exchange capacity (CEC), X-ray fluorescence (XRF), Fourier transform infrared spectroscopy (FT-IR), thermal analysis (TGA/DSC), and nitrogen adsorption at 77 K. The homoionized clay was used as an adsorbent for the removal of Cr(III) in an aqueous solution. The homoionized clay was modified with hexadecyltrimethylammonium bromide (HDTMA-Br), and the organoclay obtained was evaluated for the adsorption of Cr(VI) in aqueous solution. The XRD analysis showed that the clay from Armero-Guayabal is primarily constituted by smectite (48 wt%) followed by quartz mineral (21 wt%). The chemical analysis of bulk clay showed that the predominant oxides are SiO_2_ (55.81 wt%), Al_2_O_3_ (16.25 wt%), and Fe_2_O_3_ (7.51 wt%), and the nitrogen adsorption indicated that the bulk clay has a specific surface area of 45.1 m^2^/g. Homoionized clay and organoclay achieved Cr(III) and Cr(VI) removals greater than 85.05 ± 2.04% (pH between 3 and 4) and 82.93 ± 1.03% (pH between 3 and 5), respectively, proving the potential of these materials for the removal of heavy metals in an aqueous solution.

## 1. Introduction

The term “clay” has been defined as a naturally occurring material primarily made of fine-grained minerals (clay minerals), being generally plastic at appropriate water contents and hardening when dried or fired [1]. Phyllosilicate clay minerals are the main constituent of clays [2]. They are of great interest for the scientific community due to their potential industrial applications in many areas [2, 3]. Clay minerals are generally classified into three layer types (1:1, 2:1, and 2:1:1) based upon the number and arrangement of tetrahedral and octahedral sheets in their basic structure. These are further separated into five groups (kaolinite, mica, smectite, vermiculite, and chlorite) that differ with respect to their net charge. Smectite is the name used for a group of phyllosilicate mineral species, the most important of which are montmorillonite, beidellite, nontronite, saponite, and hectorite [4].

Montmorillonite is a 2:1 phyllosilicate that belongs to the group of smectites and it is the main component of bentonite clay. Bentonite is a material derived from the chemical alteration of a glassy material of igneous origin emitted from volcanoes (usually tuff and/or volcanic ash) [5, 6]. It was named by Wilbur C. Knight in 1898, and the name is derived from a site located in Fort Benton, United States [7]. Bentonite is currently an important material due to the large number of applications in the food industry [8, 9], paints [10], pharmaceutical industry [11, 12], and wastewater treatment [13, 14] and as a catalyst [3, 15–17].

World bentonite production has increased from 2008 to 2014, growing from 1.57 to 1.62x10^7^ metric tons according to the British Geological Survey report [18, 19]. The exploitation of bentonite in Colombia between 2009 and 2014 has risen from 8.5 to 9.0x10^3^ metric tons [19, 20], Valle del Cauca region being the main producer. Colombia has a strong pottery tradition in which clays have been used to make roof tiles, pots, and other utensils. It is necessary to explore new industrial uses because the country has an estimated 1.1x10^9^ metric tons in smectite clays deposits [21].

In Colombia, deposits with smectites resources have been identified for possible exploitation, mainly in the departments of Valle del Cauca and Tolima [22, 23]. The deposits of Valle del Cauca have been commercially exploited since the early 70's. There are several published studies on the application of bentonites from Valle del Cauca, especially modifications via pillarization with application in oxidation reactions [15, 17, 24–27]. Particularly, from a study of the Colombian Geological Survey (CGS) in 2004, it is known that deposits of smectite clays are found in Lérida, Armero-Guayabal, and Mariquita [22], all of them municipalities at the north of Tolima department (Central-west Colombia). 32 clay samples were analyzed by CGS, finding that smectite clays are present in rocks of paleogenic and neogenic volcanic origin. Besides, smectite percent in clays was estimated between 3.2% and 56.6% for the samples studied. However, bentonites from northern Tolima have been little studied, and their commercial exploitation has focused on additives for animal concentrate and cat litters.

The abundance of bentonite and its cation exchange properties make this low-cost material a strong candidate as an adsorbent for the removal of heavy metal from wastewater [28]. The chemical modification of clay mineral through the use of cationic surfactant to generate organoclays [29] has applications in environmental remediation [30]. Additionally, these materials have shown particular properties for the adsorption of many emerging pollutants, becoming a potential alternative for the remediation of micropollution [29, 31].

This study carried out the physicochemical and mineralogical characterization of Armero-Guayabal clay, and it assessed its potential use as an adsorbent for the removal of chromium from aqueous solution.

## 2. Materials and Methods

### 2.1. Bulk Clay Collection

**Figure 1:**
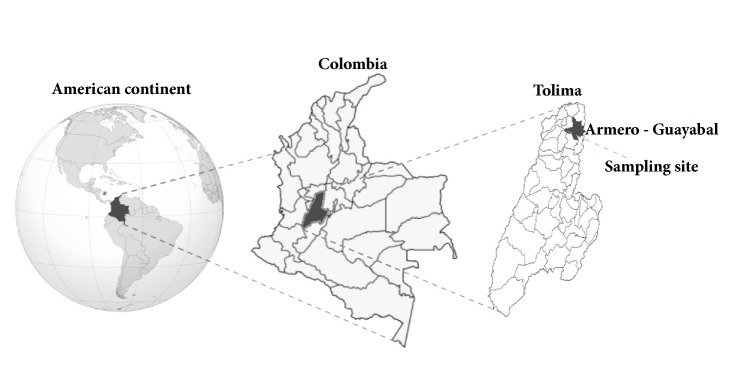
Geographic localization of clay deposit.

Bulk clay was collected from a deposit in Armero-Guayabal, a municipality in the north of the Department of Tolima, in the central-western part of Colombia (latitude 4° 58′ 4.44 N′′, longitude −74° 52′ 52.32 O′′, 366 m a.s.l.), as shown in Figure 1. The sample was collected from a mine in a zone affected by the weathering of volcanic rocks deposited in recent rock units (near the area of El Ruiz volcano) [22, 32]. The Armero-Guayabal deposit has been exploited since 2011 by the company Gea Minerales S.A.S., and it is estimated to have ore reserves of around 1.2 million tons. The samples were collected in the mine area at the same thickness of the clay layer (3 to 5 m in the vertical section), distanced by 2 m in the horizontal section. 10 bulk samples (5 kg each) were collected using hoe and shovel and then were packed in plastic bags with air tight seal. Bulk samples were dried at 105°C until reaching a constant weight. Then, they were mechanically ground, powdered in a ball mill, and passed through a 100 mesh. Equal proportion of bulk samples (1 kg) was mixed and homogenized to form a single representative sample that was used for all the tests.

### 2.2. Separation of the Clay Fraction

The particle size separation of the clay fraction was performed by gravitational sedimentation based on Stokes' Law [33, 34]. 50 g of the powder sample was suspended in 5 L of distilled water. The sample was disaggregated for approximately 20 minutes with a magnetic stirrer and transferred to a graduated cylinder to sediment gravitationally. To obtain the fraction lower than 2 *μ*m, the suspension was allowed to settle for 16 h and the first 20 cm of the suspension removed. Subsequently, the suspension was centrifuged at 5000 rpm for 15 minutes to recover the clay fraction, which was dried at 60°C, ground, and sieved in 100 mesh. This sample was used for mineralogical analysis. The clay fraction can also be called purified clay.

Although the gravimetric sedimentation is the most used method for the separation of the clay fraction (<2 *μ*m) [33], this methodology requires well diluted clay suspensions and long times making it not viable for industrial purposes. Therefore, for the adsorption tests, the clay was purified using a simple method of dispersion of ground clay in water with addition of sodium hexametaphosphate and centrifugal separation [35]. In a beaker, 50 g ground powder bulk clay was suspended in 0.6 L of distilled water with 0.5 g (NaPO_3_)_6_ and then magnetically stirred for 24 h. The supernatant of the dispersion was separated by centrifugation at 700 rpm for 2 min. The purified bentonite was obtained after centrifugation at 4700 rpm for 5 min, washed five times with distilled water, and dried at 60°C in an oven. The dried purified clay was ground and sieved in 100 mesh.

### 2.3. Mineralogical and Chemical Characterization of the Clay

The clay sample was analyzed both as random powder of the bulk material and as oriented mounts of the fraction < 2 *μ*m by using a LabX Shimadzu XRD-6000 diffractometer with Cu K*α* radiation (steps of 0.01° 2*θ* and 2 s/step). The pattern of XRD was compared with X-ray reference patterns for the identification of the different minerals, using an X'Pert HighScore Plus software. Identification of mineralogical components of smectitic type was carried out applying the protocol established by Thorez [36] and Schultz [37]. The semiquantitative mineralogical analysis was based on the signal area ratio and the intensity of clay mineral (intensity factors) [38–40]. Relative proportions of the clay minerals were normalized to 100% [37, 41].

The bulk clay dried at 60°C was ground in an agate mortar until the size of the largest particles was less than 38 *μ*m (mesh 400). The sample was introduced in the cavity of the sample holder to minimize preferred orientation of the particles. A glass slide was used to pack the sample into the cavity, just enough to create a smooth surface that would not deform during the rotation of the sample during XRD analysis [42].

Oriented mounts were prepared from the clay fraction <2 *μ*m. A few drops of the clay suspension (600 mg of clay in 5 mL of water) were smeared on a glass slide, which was left to dry at room temperature. The basic identification of the clay was made according to the position of basal reflection 001 in three patterns of X-ray diffraction: (i) natural sample, (ii) natural sample after solvation in the presence of vapor ethylene glycol for 24 h at 35°C, and (iii) glycolated natural sample after heating at 500°C for 2 h. In addition, the clay fraction <2 *μ*m was saturated with Li^+^, K^+^, and Mg^2+^ solutions and the samples subjected to XRD after the treatments described in Table 1.

Chemical analyses of the bulk and clay fraction were accomplished by X-ray fluorescence using a Magix Pro Philips PW2440 instrument, with samples prepared as pearls. Total organic carbon was estimated by Multi N/C 3100 TOC analyzer (Analytik Jena, Germany) in a horizontal high temperature oven HT1300 for solid sample analysis equipped with a nondispersive infrared detector. The calibration was made with analytical grade CaCO_3_. The calculated error for this technique was ±0.02%.

For the Foster swelling test, bulk clay and clay exchanged by sodium were sieved in a 200 mesh and dried at 105°C. Then, 2 g of dry sample was added in portions of 100 mg at 10 min intervals for 3 h to 100 mL of a sodium lauryl sulfate solution (1% w/v) contained in a 100 ml measuring cylinder. The samples were allowed to settle for 16 h before determining their volume. The test was carried out under the Colombian technical standard method NTC 2271 [43].

The cation exchange capacity (CEC) of bulk clay and clay fraction was determined with ammonium acetate. The sample was firstly ammonium exchanged; then the ammonium ions in the supernatant were deprotonated into ammonia with a sodium hydroxide solution and determined by distillation into a known amount of acid which was again titrated (Kjeldahl-method) [44].

Fourier transform infrared spectrometry (FT-IR) was recorded from samples pressed into pellets with KBr powder by using a Nicolet iS5 (Thermo Scientific).

The thermal analysis was performed in a STA 409 Netzsch instrument with a 75 cm^3^/min air flux and heating speed of 10°C/min.

Nitrogen adsorption–desorption isotherm of clay fraction was determined in a Micrometrics ASAP 2020 instrument at 77 K after outgassing the samples for 1 h at 90°C followed by 3 h at 400°C in a vacuum. The specific surface area (S_BET_) was measured by means of BET equation and the total pore volume was evaluated for nitrogen uptake at a relative pressure of 0.99. Microporous specific surface area and micropore volume were calculated using the* t*-method of the Harking-Jura equation [45, 46].

### 2.4. Homoionization of Clay

50 g of purified clay was dispersed in 5 L of 1 M solution of NaCl by shaking for about 24 h; then the supernatant was removed. This procedure was repeated to get the clay saturated with sodium. After the two exchanges with the NaCl solution, the clay obtained was washed with distilled water until reaching a conductivity lower than 10 *μ*s/cm, dried at 60°C, and finally ground and sieved in a 100 mesh.

### 2.5. Synthesis of Organoclay

The organoclay was prepared with an ion exchange reaction between the clay homoionized with sodium and an organic cationic surfactant, hexadecyltrimethylammonium bromide (HDTMA-Br). The loading of HDTMA^+^ surfactant was equivalent to 1.5 times the cation exchange capacity (CEC) for homoionized clay [47]. 20 g homoionized clay was added to 400 mL of distilled water and, to this suspension, 4.80 g of HDTMA was added. The mixture was stirred for 24 h. The organoclay was then removed from the mixture by centrifugation and then washed several times with water until being bromide-free. Finally, the organoclay was dried at 60°C, ground, and sieved in a 100 mesh.

### 2.6. Preliminary Tests of Chromium Adsorption

The effect of pH on the adsorption of Cr(III) on natural bentonite and Cr(VI) on organoclay was evaluated. The adsorption experiments were performed by using the batch method, suspending the clay (0.5 g clay or 0.2 g organoclay) in 50 mL an aqueous solution of chromium (Cr(III) or Cr(IV)) at 50 mg/L. Samples were shaken for 1 h at 200 rpm at 20°C. Standard solutions of Titrisol™ Merck® (CrCl_3_ in HCl 4.2%) and potassium dichromate (K_2_Cr_2_O_7_ 0.1 N) were used to prepare a synthetic wastewater which was diluted to 50 mg/L in order to carry out adsorption tests using the bulk clay and organoclay. The adsorbent was separated by centrifugation and supernatant solution filtered in a 0.22 *μ*m filter and then carefully transferred to glass flasks to determine the concentrations of chromium present in aqueous medium. The concentrations of chromium were measured by flame atomic absorption spectrometry (iCE 3500, Thermo Scientific, Germany), with an air-acetylene flame. All the tests were performed in triplicate.

Cr(III) and Cr(VI) adsorption isotherms were obtained by using a batch equilibration procedure. Adsorbent samples were balanced by shaking at 20°C with the solutions of chrome (concentrations ranging from 10 to 150 mg/L). The quantity of the adsorbed Cr(III) and Cr(VI) was calculated using the following equation:

(1) $$\begin{array}{c} q_{e}=\frac{\left(C_{o}-C_{e}\right)V}{m} \end{array}$$

where *q*_*e*_ is the amount of solute adsorbed in the adsorbent (mg/g), *C*_*o*_ is the initial concentration of the solute (mg/L), *C*_*e*_ is the equilibrium concentration of the solute (mg/L), V is the solution volume (L), and *m* is the mass of the adsorbent (g).

## 3. Results and Discussion

### 3.1. Mineralogical Characterization of the Clay

**Figure 2:**
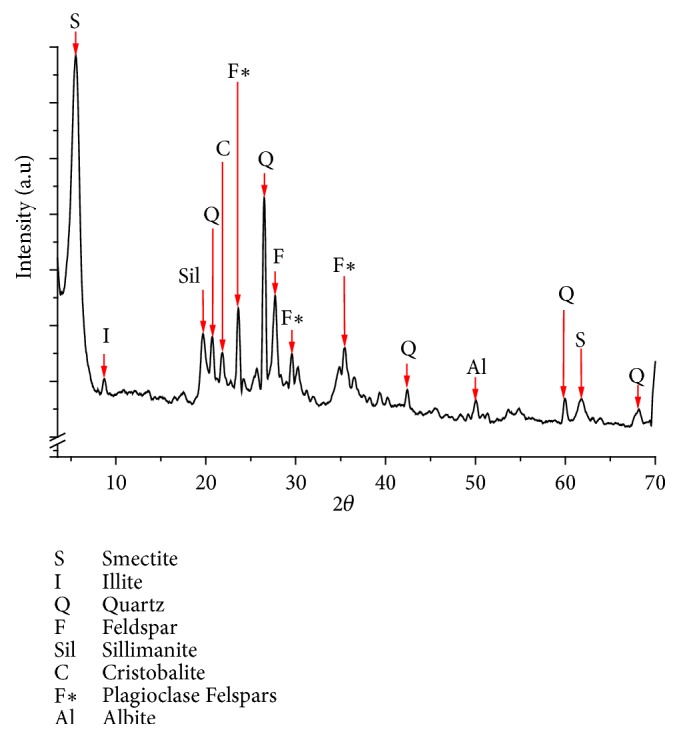
Powder X-ray diffractogram of the Armero-Guayabal clay.

The X-ray diffraction pattern of bulk clay random powder (not oriented aggregate) is shown in Figure 2. The main reflection was at 15.88 Å, which is a typical signal of smectites. The 060 reflection at 1.50 Å confirms that the clay was a dioctahedral smectite, based on this signal in the interval 1.492 Å to 1.504 Å, typical of montmorillonite [36, 48].

Armero-Guayabal clay has a considerable presence of quartz (peaks around 4.29 Å, 3.37 Å, 2.13 Å, 1.54 Å, and 1.37 Å) [49]. Felspars and sillimanite are in the sample too. For the first one, peaks were in 3.76, 3.20, and 3.02 Å [49, 50], whereas sillimanite showed one signal at 4.51 Å [49]. The sample also had contents of cristobalite (4.08 Å) [51] and illite (10.14 Å) [49].

**Figure 3:**
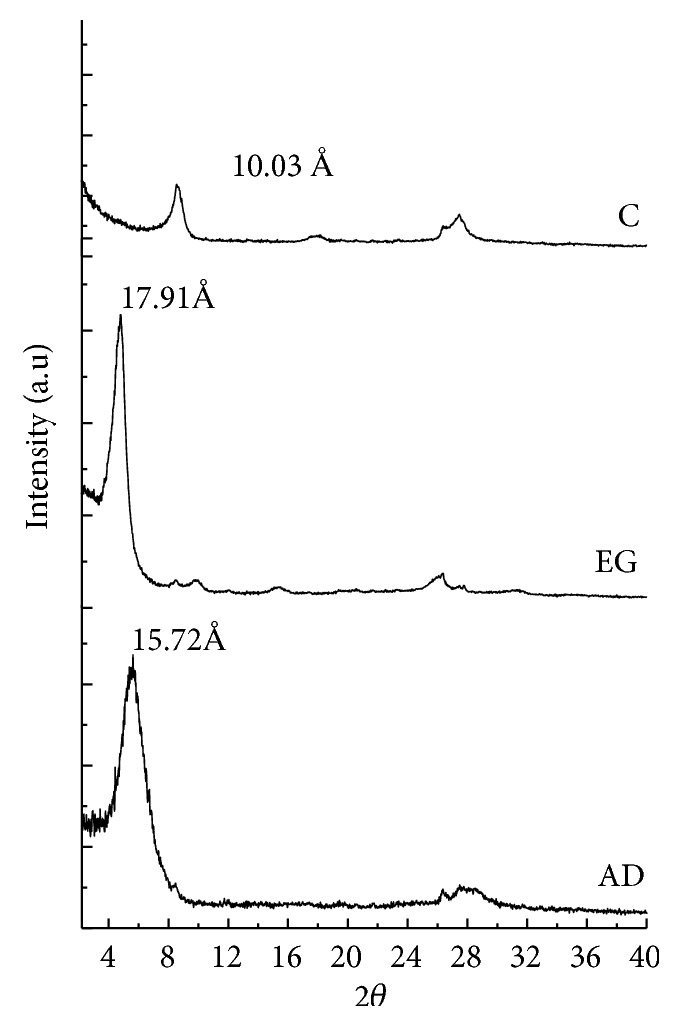
XRD pattern the clay fraction <2 *μ*m (oriented mounts): AD: air dried; EG: saturated with ethylene glycol vapor; C: calcined at 500°C.

**Figure 4:**
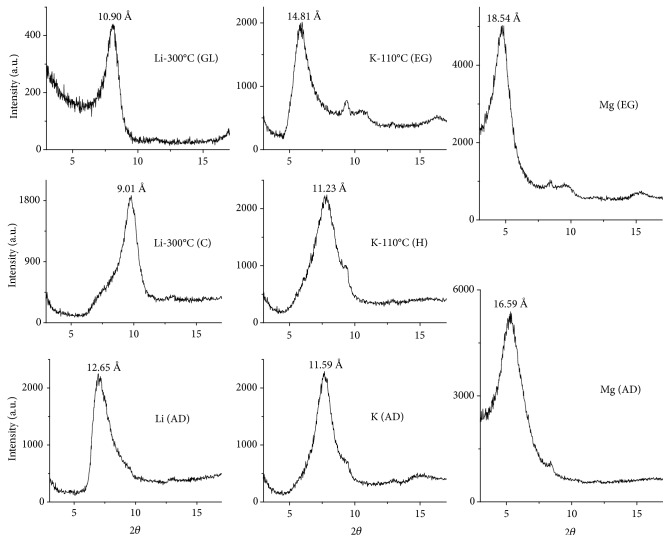
XRD pattern the clay fraction <2 *μ*m (oriented mounts) treated with solutions of Li, K, and Mg. AD: air dried; EG: ethylene glycol; GL: glycerol; H: heating; C: calcinated.

The presence of smectite was confirmed by the ethylene glycol solvation test with shifting peak of 001 from 15.72 to 17.91 Å (Figure 3). After calcination of the glycolated sample, the peak 001 shifted to 10.03 Å, indicating the clay collapse, typical behavior of a smectite [36]. The XRD patterns for the clay fractions which were saturated with Li, K and Mg solutions are shown in Figure 4.

The sample was tested with lithium for differentiating between montmorillonite and beidellite. The basis of the test was the loss of expansion and cation-exchange properties exhibited by montmorillonite but not by beidellite when it was saturated and heated with lithium. Thus, the irreversible collapse of a smectite mineral to 9.01 Å after Li-saturation and heating at 300°C is the criterion for montmorillonite identification, and it is attributed to the neutralization of the negative layer charge by migration of the lithium ions from interlayer positions to vacant octahedral sites [42, 52, 53].

K-saturated and heated clay at 110°C showed a collapse at 11.23 Å, and this behavior confirmed that montmorillonite was the main mineral in the sample [36]. The combination of high negative lattice charge and low dehydration energies for K^+^ resulted in the dehydration of potassium within these interlayer spaces and the collapse of the layer to a 10-11 Å basal spacing [54].

Additionally, ethylene glycol solvation shifted the 001 peak of Mg-saturated smectite to 17-18 Å, whereas the vermiculite 001 peak remained unchanged at 14-15 Å [55].

The semiquantitative estimation of bulk clay mineralogical composition showed that the most abundant mineral was smectite, with 48 wt%, followed by quartz (21%), plagioclase (11%), feldspar (9%), sillimanite (7%), illite, cristobalite, and albite (4%).

### 3.2. Physicochemical Characterization of the Clay

The chemical compositions of the bulk clay and fraction clay are given in Table 2. The main oxides are SiO_2_, Al_2_O_3_, Fe_2_O_3_, CaO, and MgO. The major oxides found in clay minerals are SiO_2_ and Al_2_O_3_. The SiO_2_/Al_2_O_3_ ratio has been accepted as a parameter for oxide composition of clays in order to differentiate the type of clay minerals. A recent study proposes the use of a practical chart to identify the predominant clay mineral based on oxide composition in clay soils. The average SiO_2_/Al_2_O_3_ ratio for 40 montmorillonite samples evaluated was 2.85 [56]. In this study, the SiO_2_/Al_2_O_3_ ratio was 3.43, which is higher than that reported by Sivrikaya et al. [56]. However, it is within the range found for the smectites from north of Tolima (2.70 to 3.93) [23] and three Brazilian smectites (3.14 to 4.09) [57]. Clay from Armero-Guayabal presented a reddish color, which can be associated with the high content of Fe_2_O_3_. The total organic carbon content for bulk and purified clay was 45.30 and 42.17 mg of C/kg, respectively, very low values suggesting a minimum amount of organic matter in the samples.

In accordance with the chemical composition of SiO_2_ (56.58 and 53.70 wt%), Al_2_O_3_ + Fe_2_O_3_, (23.39 and 25.12 wt%), and MgO + CaO + Na_2_O + K_2_O (8.51 and 8.63 wt%), the bulk and purified clay samples were located in the montmorillonite zone of the reported diagrams by Sivikaya et al. [56].

After the purification process the chemical composition of the bulk clay changed due to the presence of clay minerals like quartz, feldspar, sillimanite, plagioclase, and cristobalite which were removed from the sample (Figure 3), meaning changes in the chemical composition as shown in Table 2. It shows that the main exchangeable cations are Ca^2+^, Mg^2+^, K^+^, and Na^+^. The percentage of sodium increased whereas calcium, magnesium, and potassium percentage decreased for the purified clay regarding the bulk clay. Due to the purification process, sodium hexametaphosphate (NaPO_3_)_6_ (Section 2.2) was used, causing the exchangeable cations to be exchanged by sodium cations.

The cation exchange capacity (CEC) can be defined as the sum of all the cations that a mineral can associate with a certain pH. It has two origins: one is the isomorphic substitution in the tetrahedral and/or octahedral sheet of the clay mineral layer and the other is the dissociation of aluminum groups on the edges [58]. The CEC of the bulk clay and clay fraction were of 39.08 and 43.02 meq/100 g, values slightly lower than those reported for montmorillonites from the Valle del Cauca-Colombia (46.4 and 53.0 meq/100g) [15, 17] and for smectite-type clays (range within 80-150 meq/100 g) [59, 60].

The Foster swelling of the original sample (bulk clay) was estimated in 7 mL/2 g and 31 mL/2 g to the exchanged clay by sodium. According to Table 2 the bulk clay is a calcium and magnesium bentonite, and it is well known that this clay shows no swelling on hydration like the sodium bentonite [61]; for that reason the exchanged clay by sodium has a Foster swelling higher than the bulk clay sample.

**Figure 5:**
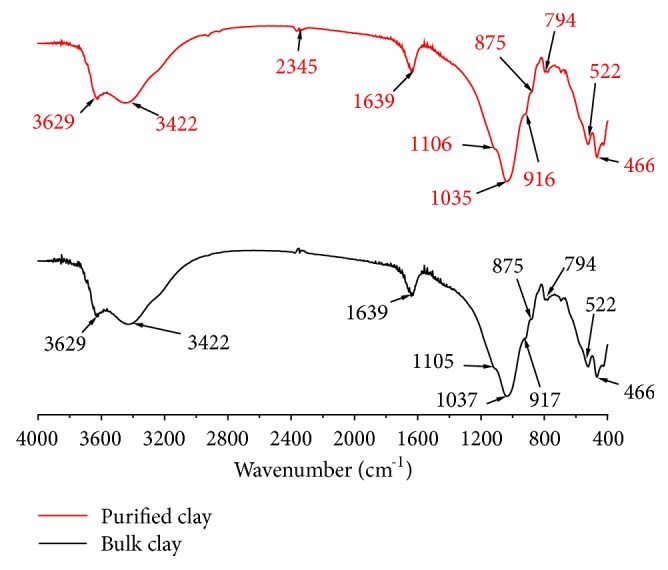
FT-IR spectra of the bulk and purified clay.

The FT-IR spectrum of the bulk and purified clay (Figure 5) shows the presence of Al-OH as stretching bands at 3629 cm^−1^ and as bending bands at 916-917 cm^−1^. The large band near 1035-1037 cm^−1^ corresponds to Si-O stretching vibration. The above signals can be considered as characteristic of a dioctahedral clay and, more precisely, a dioctahedral smectite [62, 63]. Montmorillonites contain both a tetrahedral and octahedral isomorphous substitution, Al (and occasionally Fe^3+^) for Si in the former case, and Fe^3+^ and Mg for Al in the latter. As a result of these substitutions, the crystalline order is reduced and structural imperfections arise. High-iron montmorillonites show a typical broad OH-stretching band at 3627 cm^−1^, while those with low-iron content present it at 3622 cm^−1^ [64].

The broad band centered near 3422 cm^−1^ is due to the -OH stretching mode of the interlayer water. The overlaid absorption peak in the region of 1639 cm^−1^ is assigned to the -OH bending mode of adsorbed water [65]. The band in the region of 875 cm^−1^ is due to the Si-O-Al stretching mode for montmorillonite [65, 66]. The weak band at 794 cm^−1^ corresponds to Si-O stretching vibration of quartz [62]. The bands at 522 cm^−1^ and 466 cm^−1^ are assigned to the Si–O–Al and Si–O–Si bending vibration, respectively [65–67].

**Figure 6:**
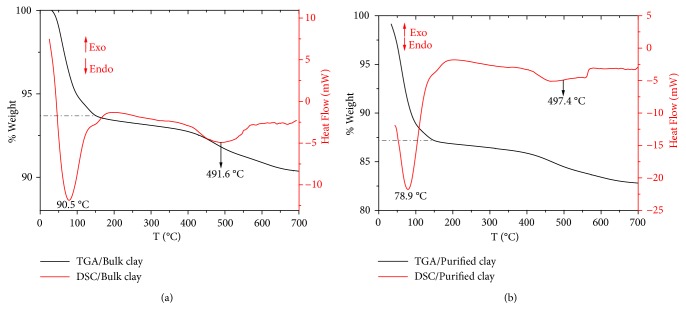
TGA/DSC curves: (a) bulk clay and (b) purified clay.

The thermogravimetric analysis (TGA) and the differential scanning calorimetry (DSC) are considered simple and accurate methods to evaluate the thermal stability and changes in the mineral under the heating process. The TGA/DSC curves for bulk and purified clays are shown in Figure 6. The first thermal step, corresponding to a reduction in mass at temperatures below 150°C, can be associated with the loss of physisorbed water molecules [68, 69] (endothermic peak centered at 90.5 and 78.5°C, respectively). This value is approximately 6.4% of the weight for bulk clay, while in a purified clay sample, it reaches up to 12.8%. In the second step, mass loss occurred due to dehydroxylation of clay (endothermic peak centered at 491.6 and 497.4°C, respectively), consistent with montmorillonites literature report [69]. The total mass loss of the bulk clay and clay fraction samples was 9.7% and 17.2%. Bulk clay showed the lowest mass loss because this material contains impurities such as quartz, which cannot physisorb water in its structure.

**Figure 7:**
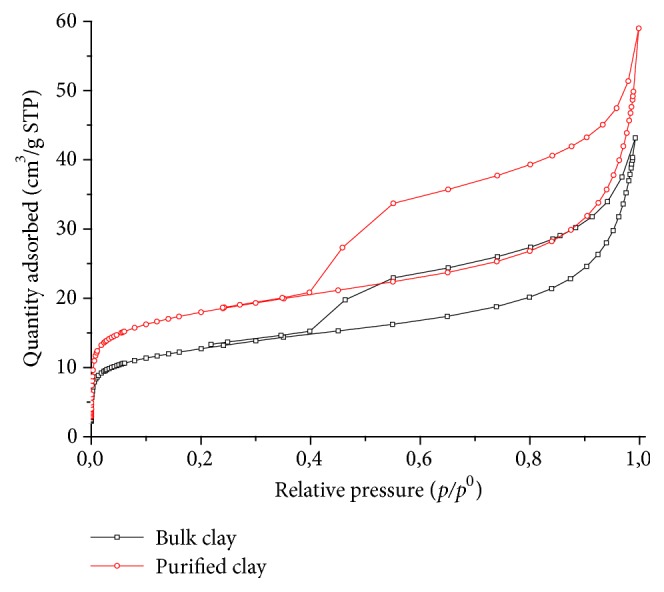
N_2_ physisorption isotherm of the bulk and purified clay.

The bulk and clay purified isotherms are shown in Figure 7. They were classified as a type IVa and their hysteresis loops are H3. The initial part of isotherm (p/p° < 0.41), corresponding to the monolayer region, could be ascribed to physique adsorption at the surface of the clay. There are two distinctive features of the type H3 loop: (i) the adsorption branch resembles a type II isotherm and (ii) the lower limit of the desorption branch is normally located at the cavitation-induced p/p^0^. The loop of this type is given by nonrigid aggregates of plate-like particles [46].

The specific surface area was 45.1 m^2^/g and 62.9 m^2^/g for bulk and purified clay, respectively. The pore volume at* p/p*° = 0.99 is of 0.0654 cm^3^/g for bulk material and 0.0853 cm^3^/g for purified clay. The *t* plot method was used to determine the specific area and the volume developed by micropores of the samples. These values are 8.7 m^2^/g and 0.0031 cm^3^/g for bulk clay and 16.5 m^2^/g and 0.0058 cm^3^/g for clay fraction, respectively. The specific surface area of these materials is fundamentally the external surface, characteristic of a structure of closed sheets, attributed to the heterogeneous arrangement of the aluminosilicate sheets [1].

### 3.3. XRD of Organoclay

**Figure 8:**
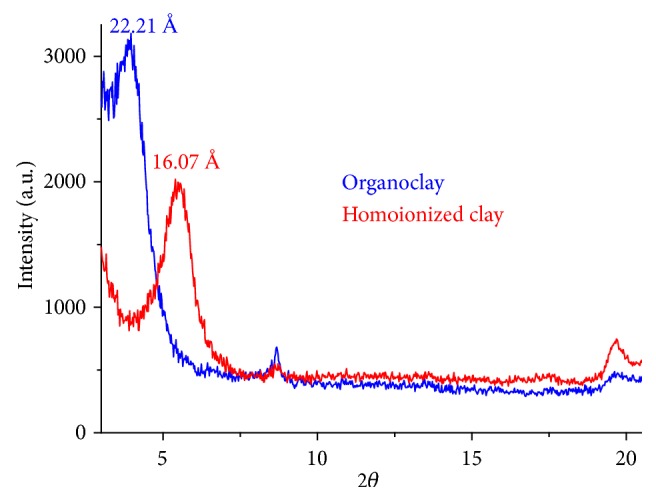
XRD of homoionized clay and organoclay.

The shift of the d_001_ signal from 16.02 Å, for the homoionized clay, to 22.21 Å, for the organoclay (see Figure 8), is associated with the incorporation of the HDTMA cation in a pseudo-trilayer/paraffinic arrangement (d_001_> 22.0 Å, aliphatic chains oblique to the basal surface) [70]. Variation between the basal spacing d_001_ and the concentration of the surfactant has been found [71, 72]. Upon increasing the surfactant loading (HDTMA-Br), the interlayer spacing of organoclays gradually increased. There is a lateral monolayer arrangement in 0.25 CEC and 0.5 CEC. The basal spacing at 18.2 Å with a shoulder of 22.0 Å indicates a lateral bilayer arrangement and the d_001_ spacing of 22.0 Å at 2.0 CEC suggests a pseudotrimolecular layer arrangement [72].

### 3.4. Preliminary Tests of Chromium Adsorption

**Figure 9:**
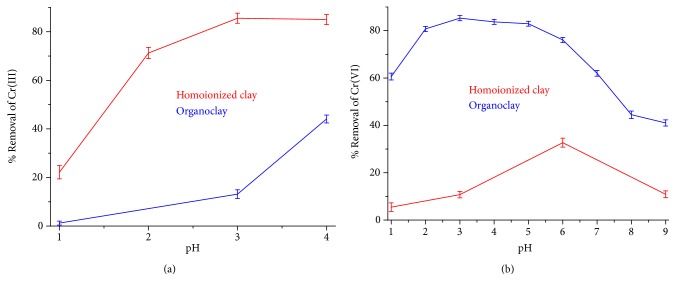
Adsorption of (a) Cr(III) and (b) Cr(VI) on homoionized clay and organoclay. The error bars represent the standard deviation of measurements of Cr in three adsorption tests.

Homoionized clay and organoclay samples were used to evaluate the effect of pH on the adsorption of Cr(III) and Cr(VI) ions from an aqueous solution (see Figure 9). The homoionized clay removed trivalent chromium by exchanging the Na^+^ compensation ions by Cr^3+^ ions. The organoclay removed hexavalent chromium by means of an anionic exchange mechanism of the counter-ion bromide present in the molecule of HDTMA-Br having been exchanged by ions Cr_2_O_7_^2-^ and HCrO_4_^−^ [73]. The organic surfactant was fixed to the interlayer of bentonite by van der Waals hydrophobic interactions [30].

At pH 3 and 4, homoionized clay achieved a Cr(III) removal greater than 85.05 ± 2.04%, while organoclay had the highest removal (44.09 ± 1.62%) at pH 4. No tests were performed at pH values greater than 4.8 because removal of Cr(III) was promoted by precipitation and not by cation exchange with clay [73]. Removals of Cr(VI) greater than 82.93 ± 1.03% were obtained with the organoclay at pH between 3 and 5, while homoionized clay achieved a removal of 32.69 ± 1.89% at pH 6.

**Figure 10:**
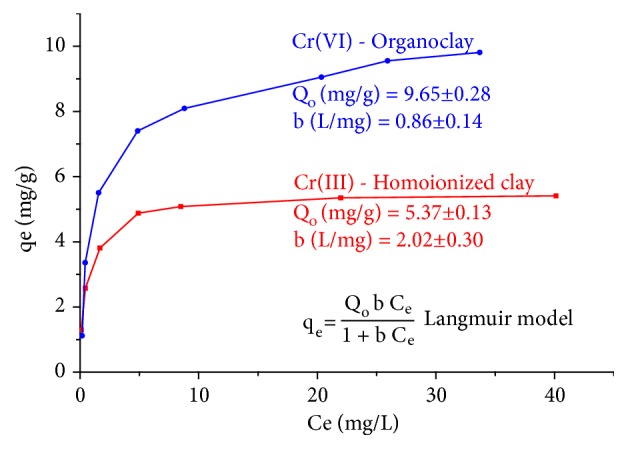
Adsorption isotherms of Cr(III) and Cr(VI) at 20°C and V=50 mL in homoionized clay (pH 3.5, 0.96 g adsorbent) and organoclay (pH 3.4, 0.44 g adsorbent).

In Figure 10, the adsorption isotherms of Cr(III) and Cr(VI) are presented. Data of isotherms (*q*_*e*_ vs *C*_*e*_) were adjusted to the Langmuir model by means of a nonlinear regression analysis. In both cases, the adjustment of data had an R^2^ of 0.98.

The maximum adsorption capacity of Cr(III) in homoionized clay was 5.37 mg/g, value slightly higher than that obtained with a bentonite from Gaomiaozi, China (4.68 mg/g) [74]. For Cr(VI), a maximum adsorption capacity of 9.65 mg/g was obtained, a value higher than that obtained for this metal at pH 4 in an organobentonite (8.08 mg/g) [75]. When activated carbon is used as an adsorbent for the removal of Cr(III) and Cr(VI), *Q*_*o*_ values of 2.33 (pH 4, 25°C) [76] and 15.07 mg/g (pH 5.38, 20°C) [77], respectively, have been reported.

## 4. Conclusions

The bulk clay of Armero-Guayabal is composed mainly of SiO_2_, Al_2_O_3_, and Fe_2_O_3_, in a proportion of 56.58, 15.88, and 7.51 wt%, respectively. These values are in the range established for montmorillonites, which is the main mineral component of bentonites. The mineralogical composition of the bulk clay showed that the most abundant mineral is smectite (48 wt%). The introduction of hexadecyltrimethylammonium cations (HDTMA^+^) in the interlaminar spacing of the homoionized clay produced an increase in the basal spacing (d_001_) of the clay, going from 16.70 to 22.21 Å. This result demonstrates the modification potential of bentonite-type clay due to its high cation exchange capacity. Homoionized clay and organoclay achieved Cr (III) and Cr (VI) removals greater than 85.05 ± 2.04% (pH between 3 and 4) and 82.93 ± 1.03% (pH between 3 and 5), respectively, demonstrating the potential of these materials as adsorbents for the removal of heavy metals in an aqueous solution. This work was intended to become useful for future studies related to the use of this Colombian clay type bentonite for industrial purposes as an adsorbent or as a substrate to obtain nanostructured materials.

## Figures and Tables

**Table 1:** Further tests for detailed identification of the clay [36].

**Treatment 1**	**Treatment 2**	**Treatment 3**
Saturation with 0.5 M LiCl, 24 h, 25°C	Saturation with 0.5 M KCl, 24 h, 25°C	Saturation with 0.5 M MgCl_2_, 24 h, 25°C

Calcination at 300°C, 2 h	Heating a 100°C, 2 h	Saturation with vapour ethylene glycol, 24 h, 35°C
Saturation with vapour glycerol, 24 h, 35°C	Saturation with vapour ethylene glycol, 24 h, 35°C	

**Table 2:** Chemical composition of the bulk and purified clay used in this study compared with literature data and previous studies.

Compound	Chemical Composition (wt%)			
	Bulk clay	Purified clay	Montmorillonites*∗* [56]	Smectites*∗∗* [22]
SiO_2_	56.58	53.70	57.68	57.84
Al_2_O_3_	15.88	16.79	20.22	16.93
Fe_2_O_3_	7.51	8.33	3.17	5.84
CaO	3.43	2.50	1.20	1.46
MgO	2.24	2.46	4.22	1.51
K_2_O	1.73	1.45	0.44	1.36
Na_2_O	1.11	2.22	0.98	0.94
TiO_2_	0.70	0.68	0.24	0.52
MnO	0.13	0.11	1.74	0.06

*∗*: average of 40 montmorillonite samples [56].

*∗∗*: average of 32 smectite samples located in the north of Tolima [22].

## Data Availability

All the data were included in the article through figures and tables.
